# eHealth Literacy Mediating Social Support and Technology Acceptance Among Patients With Chronic Illnesses: A Cross‐Sectional Study

**DOI:** 10.1111/jan.70207

**Published:** 2025-09-08

**Authors:** Lian‐Shin Shiu, Yu‐Shan Huang, Chieh Yu Liu, Yu‐Shan Cheng, Yu‐Chi Chen

**Affiliations:** ^1^ International Priority Care Center Taiwan Adventist Hospital Taipei Taiwan; ^2^ Department of Nuraing, College of Nursing National Yang Ming Chiao Tung University Taipei Taiwan; ^3^ Kaillan Group Practice Clinic Yilan Taiwan; ^4^ Institution of Community Health Care, College of Nursing National Yang Ming Chiao Tung University Taipei Taiwan; ^5^ School of Nursing, The University of Texas at Austin Austin Texas USA; ^6^ Institute of Clinical Nursing, College of Nursing National Yang Ming Chiao Tung University Taipei Taiwan

**Keywords:** chronic illnesses, culturally tailored intervention, eHealth literacy, eHealth technology acceptance, health behaviour, nurses, nursing practice, patient engagement, self‐management, social support

## Abstract

**Aim:**

To examine the relationships among social support, eHealth literacy and eHealth technology acceptance among patients with chronic illnesses, and investigate whether eHealth literacy plays a mediating role.

**Design:**

A cross‐sectional correlational study.

**Methods:**

A total of 202 patients with chronic illnesses were recruited from outpatient clinics and communities in Taiwan. Data were collected via structured questionnaires and analysed using SPSS and PROCESS macro with 1000 bootstrap samples.

**Results:**

eHealth literacy was the strongest predictor of technology acceptance. Although social support was positively associated with eHealth literacy, it did not directly predict technology acceptance after controlling for eHealth literacy, indicating a full mediation effect.

**Conclusions:**

eHealth literacy is a crucial mechanism through which social support influences health technologies acceptance. Interventions to improve eHealth literacy, particularly those integrated with social support strategies based on different cultural backgrounds, enhance digital engagement among chronic illnesses.

**Implications for Profession and/or Patient Care:**

Healthcare professionals and policy‐makers should design literacy‐sensitive interventions that leverage social networks and involve significant others to promote meaningful eHealth engagement in disease management.

**Impact:**

eHealth literacy fully mediates the relationship between social support and eHealth technology acceptance, proving that social support alone does not directly increase adoption without improving eHealth literacy. eHealth literacy is the strongest predictor of eHealth technology acceptance, emphasising its central role in bridging the gap between social support and eHealth engagement.

**Reporting Method:**

This study followed the STROBE checklist guideline.

**Patient or Public Contribution:**

No patient or public involvement.

**Summary**

- What Does This Paper Contribute to the Wider Global Clinical Community?
- Identifies eHealth literacy as a critical mediator—not just an associated factor—linking social support to acceptance of eHealth technologies among patients with chronic illnesses.
- Highlights the necessity of culturally sensitive interventions, showing that social support alone is insufficient to drive technology acceptance, particularly in collectivist societies.
- Provides evidence to guide the development of integrated strategies that enhance both eHealth literacy and social support, supporting improved adoption and sustained use of digital health solutions globally.

## 1 Introduction

Chronic illnesses are the leading contributors to global morbidity and mortality, accounting for nearly 74% of all deaths worldwide (World Health Organization 2024). With increasing life expectancy and a rising prevalence of chronic diseases, the burden on healthcare systems and national economies has intensified. By 2030, chronic illnesses are projected to cause an estimated global economic loss of USD 47 trillion (Hacker 2024), underscoring the urgent need for sustainable management approaches that improve patient outcomes while containing healthcare costs (Budreviciute et al. 2020; Centers for Disease Control and Prevention 2024).

Digital healthcare has emerged as a mainstream global strategy to advance universal health coverage and sustainable development goals (World Health Organization 2021). eHealth technologies—including digital platforms for remote monitoring, real‐time feedback and virtual health communication in chronic conditions such as diabetes, cardiovascular disease and functional impairments—hold considerable potential to optimise disease management by empowering patients to enhance their self‐management capabilities, supporting clinicians in delivering timely, personalised and proactive care, and alleviating the long‐term burden on healthcare systems through improved resource utilisation and reduced hospitalisations (Adeghe et al. 2024; Chhikara and Verma 2024). However, despite increasing availability, real‐world adoption remains low (Lee et al. 2022; Madrigal and Escoffery 2019), suggesting that patient acceptance, rather than technological accessibility, constitutes a critical barrier (Amagai et al. 2022). Reported obstacles include low digital motivation, perceived complexity and inadequate eHealth literacy (Jakob et al. 2022; Walle et al. 2023), all of which can limit sustained engagement with digital health interventions (Wang and Ku 2020).

eHealth literacy has emerged as a pivotal determinant of successful engagement with digital health technologies. Defined as the ability to seek, appraise and apply health information from digital sources for informed health management, eHealth literacy integrates the competencies of health literacy and digital literacy (Kayser et al. 2018; Norgaard et al. 2015; Li et al. 2023). According to the eHealth literacy framework (Norgaard et al. 2015), eHealth literacy extends beyond basic technical skills to encompass confidence, critical interpretation and personalised application of digital health information. It reflects individuals' capacity to confidently engage with digital technologies and incorporate health information into daily care decisions and overall health management (Campanozzi et al. 2023; Kayser et al. 2018; Norgaard et al. 2015). Therefore, eHealth literacy enables patients to navigate, evaluate and apply health‐related information from digital sources or platforms, including interactions with AI‐enabled services, which are increasingly essential for achieving health equity and meaningful participation in smart healthcare (Campanozzi et al. 2023; Van Der Vaart and Drossaert 2017). Higher eHealth literacy is associated with increased confidence, empowerment and active chronic disease self‐management, ultimately leading to better health outcomes (Shiu et al. 2023). Conversely, low eHealth literacy relates to challenges in trust and utilisation of digital tools, resulting in disengagement even when platforms are accessible (Amagai et al. 2022; Jakob et al. 2022). Thus, equitable and effective digital health care requires not only access to technology but also proactive support to build patients' eHealth literacy competencies (Cheng et al. 2024).

Social support is recognised as a key enabler of eHealth technology acceptance, particularly among older adults and patients managing chronic illnesses (Reiners et al. 2019). Support from family members, peers, healthcare professionals and community networks facilitates technology adoption by providing practical assistance, encouragement and emotional reinforcement (Choi 2020). This influence is especially strong in collectivist cultures—such as many East Asian societies—where health‐related decisions are often made in consultation with trusted social figures. Individuals in these contexts tend to rely on family or healthcare professionals for guidance before adopting new technologies, whereas individualistic cultures place greater emphasis on personal autonomy (Chu et al. 2024; Li et al. 2021). Although social support can encourage patients to initially try eHealth tools, its role in sustaining long‐term use is complex. While initial adoption may be socially driven, continued engagement often depends on the individual's confidence and competence in navigating digital platforms (Chen et al. 2024; Jakob et al. 2022).

In this regard, eHealth literacy may serve as a critical mediating factor between social support and eHealth technology acceptance. While social support can enhance exposure to and motivation for using digital health tools, meaningful and sustained engagement ultimately depends on adequate digital skills and confidence (Cheng et al. 2024; Lee et al. 2023). The influence of social support on technology acceptance is complex: strong social networks may promote initial adoption but do not necessarily guarantee continued use (Amagai et al. 2022). Patients lacking confidence in their eHealth literacy may discontinue use of digital health tools despite external encouragement (Chen et al. 2024). This suggests that social support enhances eHealth literacy, which in turn increases patients' willingness and ability to adopt and sustain use of eHealth solutions (Cheng et al. 2024; Shiu et al. 2023). Although prior studies have examined the independent effects of social support and eHealth literacy on engagement with digital health interventions (Lee et al. 2024), few have addressed their interactive effects, and the mediating role of eHealth literacy remains underexplored—particularly in non‐Western contexts. Clarifying this mechanism is essential for understanding how social and individual‐level factors jointly influence eHealth technology adoption, especially among populations with chronic illnesses where sustained self‐management is critical (Campanozzi et al. 2023; Rahdar et al. 2023; Yumei et al. 2024).

This study aims to examine the relationships among social support, eHealth literacy and acceptance of eHealth technologies in adults living with chronic illnesses, and to determine whether eHealth literacy mediates the association between social support and technology acceptance. By elucidating this mediating mechanism, the study provides empirical evidence to inform the design of culturally tailored interventions that integrate eHealth literacy enhancement with social support strategies, thereby promoting sustained and meaningful engagement with digital health technologies in chronic disease management. The findings also advance understanding of the social and cognitive enablers of digital health participation, offering practical guidance for developing targeted interventions to improve adoption and long‐term use of eHealth solutions in chronic illness care.

## 2 Methods

### 2.1 Design

We used a cross‐sectional design to explore the predictor of eHealth technology acceptance and the role of eHealth literacy. The Strengthening the Reporting of Observational Studies in Epidemiology (STROBE) guidance was adopted for reporting.

### 2.2 Setting and Procedure

Participants were recruited using purposive sampling from outpatient clinics in endocrinology, nephrology, cardiology, family practice departments in hospitals, as well as from community health centres across urban and rural regions of Taiwan. This approach ensured representation from diverse healthcare settings and patient populations. A structured questionnaire was used to collect data on patient demographics, eHealth literacy, social support and acceptance of eHealth technology.

Taiwan operates a universal, single‐payer National Health Insurance (NHI) system that covers over 99% of the population, providing equitable access to healthcare from large medical centres to local community clinics and health centres. Under NHI chronic disease management requirements, patients are scheduled for follow‐up visits approximately every 3 months for consultation and evaluation.

To identify eligible participants, clinic and health centre staff reviewed appointment lists 1 day in advance. On the day of the visit, trained research assistants or nursing staff approached potential participants in the waiting area, explained the study purpose, procedures and confidentiality measures in person, and addressed any questions. Those who agreed provided written informed consent and completed the questionnaire independently in a private setting. Personalised, face‐to‐face recruitment by trusted healthcare professionals in familiar care settings likely contributed to the high response rate.

### 2.3 Inclusion and Exclusion Criteria

Eligibility criteria include: (1) patients diagnosed with chronic kidney disease, diabetes and chronic cardiovascular disease; (2) aged 20 years or above; and (3) able to speak Mandarin or Taiwanese. Exclusion criteria include: (1) severe mental illness or disorder; (2) long‐term paralysis or being bedridden; (3) gestational diabetes.

### 2.4 Data Collection

#### 2.4.1 Personal Characteristics

Data collected included information on personal characteristics, including age, gender, educational level, occupational status, marital status, religion, perceived severity of disease and perceived health status.

#### 2.4.2 Social Support

Social support was measured using the Chronic Illness Resources Survey, originally developed by Glasgow et al. (2000), to assess the availability and adequacy of support for chronic disease self‐management across eight domains (personal support, family and friends, healthcare team, neighbourhood, community, media and policy, community organisations and workplace support). The original 64‐item scale (56 items scored) used a 5‐point Likert scale (1 = not at all, 5 = a great deal), with total scores ranging from 56 to 280, and demonstrated high internal consistency reliability (Cronbach's *α* = 0.90) (Glasgow et al. 2000). To address practical constraints, the original research team developed a validated 21‐item shortened version, preserving the eight original dimensions while reducing redundancy.

The 21‐item version retains strong psychometric properties, with test–retest reliability of *r* = 0.83 at 1 month and *r* = 0.65 at 4 months, Cronbach's *α* = 0.79, and a high correlation with the full scale (*r* = 0.94) (Glasgow et al. 2000). Our study adopted this validated 21‐item version. The Chinese version, translated and validated by Liao (2015), showed a Content Validity Index of 0.92 and Cronbach's *α* of 0.841 among patients with early chronic kidney disease.

#### 2.4.3 eHealth Literacy

eHealth literacy was assessed using the eHealth Literacy Questionnaire developed by Kayser et al. (2018) based on the eHealth Literacy Framework. The scale consists of 35 items covering seven core dimensions, including: (1) Using technology to process health information, (2) Understanding health concepts and language, (3) Ability to actively engage with digital services, (4) Feel safe and in control, (5) Motivated to engage with digital services, (6) Access to digital services, (7) Digital services that suit individual needs. Each item is rated on a 4‐point Likert scale (1 = strongly disagree, 4 = strongly agree), with total scores ranging from 35 to 140. The Chinese version of the eHealth literacy questionnaire, validated by Chen et al. (2022) among patients with chronic diseases in Taiwan (Data S1), demonstrated strong psychometric properties, showing good internal consistency reliability (Cronbach's *α* = 0.75–0.95) and high content validity.

#### 2.4.4 eHealth Technology Acceptance

eHealth technology acceptance was measured using the eHealth Acceptance Questionnaire, adapted from the Technology Acceptance Model by Davis et al. (1989) and modified for healthcare applications by Huang (2019) (Data S1). The 21‐item scale assesses four key dimensions: perceived usefulness, perceived ease of use, attitude toward using eHealth, and behavioural intention to use eHealth technologies. Each item was rated on a 4‐point Likert scale (1 = strongly disagree, 4 = strongly agree), with higher scores reflecting greater eHealth acceptance. The questionnaire demonstrated excellent internal consistency reliability (Cronbach's *α* = 0.991) and strong content validity (CVI = 0.88) in previous studies.

### 2.5 Data Analysis

All statistical analyses were conducted using SPSS version 29 and SPSS PROCESS version 4.2 (Hayes 2022). Descriptive statistics were used to summarise demographics, eHealth literacy, social support and eHealth technology acceptance, including numerical distribution, percentages, means and standard deviations. Inferential analyses used independent *t*‐tests, one‐way ANOVA and Pearson's correlation coefficient to explore associations between variables such as age, gender, education, occupation, marital status, religion, perceived severity of disease, perceived health status, eHealth literacy, social support and eHealth technology acceptance. Multiple regression analysis was employed to identify significant predictors of eHealth technology acceptance, and SPSS PROCESS Model 4 was used to assess the mediating effect of eHealth literacy on the relationship between social support and eHealth technology acceptance. To test the indirect effect, bootstrapping (1000 resamples) was performed to generate a 95% confidence interval, confirming the statistical significance of the mediation effect (Hayes 2022).

### 2.6 Ethical Considerations

This study was conducted in accordance with the ethical principles of the Declaration of Helsinki (World Medical Association 2013) and the national and institutional research ethics guidelines of Taiwan (Ministry of Health and Welfare 2014, 2019). The study protocol was approved by the Human Research and Ethics Committee of National Yang‐Ming University (YM106120E; February 10, 2018) and the Institutional Review Board (IRB) of SHIN KONG WU HO‐SU Memorial Hospital (20190104R; March 14, 2019).

Prior to data collection, all participants received a detailed explanation of the study objectives, procedures, potential risks and benefits, and were encouraged to ask questions. Those who agreed to participate provided written informed consent. Participants were assured that their participation was voluntary and that they could withdraw from the study at any time without consequences. All collected data were anonymised. To protect confidentiality, all personally identifiable information was removed from the dataset, and data were stored securely with access limited to authorised research personnel.

## 3 Results

### 3.1 Participant Characteristics

A total of 202 eligible participants were approached for the study. Among them, 17 (8.42%) refused to participate, and 6 (2.97%) were excluded due to incomplete questionnaire data, leaving 169 valid responses (83.66%). Most participants were male (55.36%), married (77.51%), and employed (60.95%). The mean age was 58.48 years (SD = 13.54), with 78.6% of participants aged between 41 and 74 years. The largest age group was 65 to 74 years (27.20%), followed by 55 to 64 years (26.00%) and 41 to 54 years (25.40%). Regarding educational background, 40.96% had a high school or junior college education, and 18.07% held a college or university degree. Buddhism was the most common religious affiliation (39.02%). In terms of severity of disease, most participants perceived their disease severity as moderate (58.18%), followed by mild (35.76%) and severe (6.06%). Regarding self‐rated health, the majority of participants rated their health status as average (58.33%), while 25.00% rated it as good (Table 1).

**TABLE 1:** Participant characteristics in chronic illnesses.

	*N* (%)	Mean (SD)	Min	Max
Age	169	58.48 (13.54)	21	86
21–40 (1)	19 (11.2)			
41–54 (2)	43 (25.4)			
55–64 (3)	44 (26.0)			
65–74 (4)	46 (27.2)			
≥ 75 (5)	17 (10.1)			
Gender	168			
Male	93 (55.36)			
Female	75 (44.64)			
Education	166			
Primary school or below (1)	35 (21.08)			
Junior high school (2)	33 (19.88)			
High school or junior college (3)	68 (40.96)			
College or university or above (4)	30 (18.07)			
Occupation	169			
No	66 (39.05)			
Yes	103 (60.95)			
Marital status	169			
Single	38 (22.49)			
Married	131 (77.51)			
Religion	164			
No	36 (21.95)			
Buddhism	64 (39.02)			
Taoism	44 (26.83)			
Other	20 (12.20)			
Perceived severity of disease	165			
Mild	59 (35.76)			
Moderate	96 (58.18)			
Severe	10 (6.06)			
Perceived health status	168			
Poor	28 (16.67)			
Average	98 (58.33)			
Good	42 (25.00)			

An analysis of eHealth technology acceptance scores across demographic groups showed significant differences by age (*F* = 5.885, *p* < 0.001), education (*F* = 4.597, *p* = 0.004), occupation (*F* = 14.499, *p* < 0.001), religion (*F* = 2.808, *p* = 0.041), and perceived health status (*F* = 3.901, *p* = 0.022). Participants aged 41–54 years had significantly higher eHealth acceptance scores than those aged 55–64 years (*p* < 0.05) and ≥ 75 years (p < 0.05). Similarly, participants with higher educational attainment had significantly greater eHealth acceptance than those with only primary education (*p* < 0.05) (Table 2).

**TABLE 2:** Correlation analysis between participant characteristics and eHealth technology acceptance.

	Social support		eHealth literacy		eHealth technology acceptance	
	Mean (SD) score	*F*/*t*/*r*/Scheffe/Games‐Howell	Mean (SD) score	*F*/*t*/*r*/Scheffe/Games‐Howell	Mean (SD) score	*F*/*t*/*r*/Scheffe/Games‐Howell
Age		2.999*		5.508**		5.885***
21–40 (1)	84.11 (19.02)		108.95 (11.51)	(1) > (4)	63.26 (8.83)	
41–54 (2)	93.21 (14.63)		109.56 (13.50)	(2) > (4)	65.44 (8.14)	(2) > (3)
55–64 (3)	92.93 (13.62)		101.70 (13.57)		58.68 (8.32)	
65–74 (4)	92.04 (16.51)		101.89 (13.42)		59.89 (8.28)	
≥ 75 (5)	81.18 (16.64)		94.12 (13.33)		55.76 (10.08)	(2) > (5)
Gender		1.273		0.462		0.0262
Male	92.02 (16.00)		104.41 (13.96)		61.20 (8.99)	
Female	88.85 (16.08)		103.41 (13.80)		60.84 (8.94)	
Education		0.684		6.973**		4.597**
Primary school or below (1)	88.86 (16.33)		97.14 (15.09)	(1) < (4)	56.46 (9.78)	(1) < (4)
Junior high school (2)	90.18 (16.29)		99.61 (17.49)	(2) < (4)	60.00 (11.57)	
High school or junior college (3)	89.99 (14.62)		105.74 (8.85)	(1) < (3)	62.28 (6.30)	(1) < (3)
College or university or above (4)	94.13 (18.98)		110.17 (13.15)		63.33 (7.93)	
Occupation		4.132*		15.542***		14.499***
No	87.48 (15.01)		98.73 (13.85)		57.79 (8.01)	
Yes	92.57 (16.40)		107.06 (13.11)		62.98 (9.04)	
Marital status		0.283		0.768		0.768
Single	87.74 (16.85)		104.87 (14.30)		62.08 (8.44)	
Married	91.41 (15.75)		103.50 (13.91)		60.63 (9.15)	
Religion		1.758		4.646**		2.808*
No	89.08 (18.73)		108.22 (14.09)		62.39 (9.12)	
Buddhism	89.36 (14.45)		100.66 (11.64)		58.66 (8.93)	
Taoism	88.93 (15.51)		100.70 (14.42)		60.50 (6.52)	
Other	97.90 (16.33)		110.00 (15.18)		64.25 (9.03)	
Perceived severity of disease		0.651		1.229		0.968
Mild	90.44 (14.91)		104.15 (12.08)		61.46 (7.13)	
Moderate	90.02 (17.37)		103.05 (14.73)		60.39 (9.28)	
Severe	95 (9.25)		110.30 (17.40)		64.30 (15.09)	
Perceived health status		4.612*		1.200		3.901*
Poor	87.68 (14.85)		102.64 (13.37)		61.32 (9.31)	
Average	88.62 (15.28)		102.89 (14.12)		59.50 (8.63)	
Good	96.88 (17.23)		106.69 (14.11)		64.05 (9.11)	
Social support	**—**	**—**	0.38**	**—**	**—**	0.278***
eHealth literacy	**—**	0.38**	**—**	**—**	**—**	0.776***

* *p* < 0.05.

** *p* < 0.01.

*** *p* < 0.001.

### 3.2 Predicting Factors for eHealth Technology Acceptance

A multiple regression analysis was conducted to examine the predictors of eHealth technology acceptance (Table 3). The model explained 61.1% of the variance in eHealth technology acceptance (*F* = 36.864, *p* < 0.001, adjusted *R* ^2^ = 0.611). Among the independent variables, eHealth literacy was the strongest predictor of eHealth technology acceptance (*β* = 0.782, *t* = 13.213, *p* < 0.001). Other demographic variables, including age, education, occupation, religion and perceived health status, did not correlate significantly with eHealth technology acceptance (*p* > 0.05). Additionally, social support did not have a significant direct relationship with eHealth technology acceptance (*β* = −0.075, *t* = −1.357, *p* = 0.177).

**TABLE 3:** Predicted factors of eHealth technology acceptance.

Variable	*B*	SE	*β*	*t*	*p*
(Constant)	9.053	4.423		2.047	0.042
Age	0.021	0.426	0.003	0.050	0.960
Education	0.104	0.477	0.012	0.219	0.827
Occupation	0.660	1.995	0.037	0.663	0.508
Religion	0.671	0.466	0.072	1.441	0.152
Perceived health status	1.219	0.740	0.087	1.646	0.102
Social support	−0.041	0.030	−0.075	−1.357	0.177
eHealth literacy	0.494	0.037	0.782	13.213	< 0.001

*Note:* *R* ^2^ = 0.628; adjusted *R* ^2^ = 0.611; *F* = 36.864, *p* < 0.001.

### 3.3 The Mediating Role of eHealth Literacy in the Relationship Between Social Support and eHealth Technology Acceptance

**FIGURE 1:**
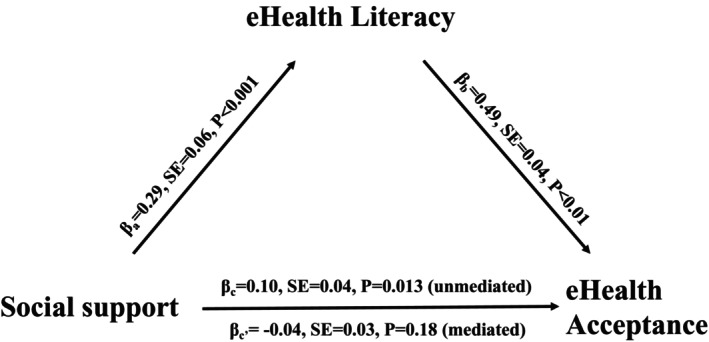
The full mediating effect of eHealth literacy in the relationship between social support and technology acceptance.

The mediation analysis confirmed that eHealth literacy fully mediates the relationship between social support and eHealth technology acceptance (Figure 1). The results show that social support was significantly associated with eHealth literacy (path a: *β* = 0.29, SE = 0.06, *p* < 0.001), indicating that individuals with greater social support tend to exhibit higher levels of eHealth literacy. Furthermore, eHealth literacy was significantly positively related to eHealth technology acceptance (path b: *β* = 0.49, SE = 0.04, *p* < 0.01), suggesting that higher eHealth literacy levels are linked to greater acceptance of eHealth technologies (Data S2).

Before accounting for eHealth literacy, social support demonstrated a significant positive association with eHealth technology acceptance (path c: *β* = 0.10, SE = 0.04, *p* = 0.013). However, after including eHealth literacy in the model, the direct effect of social support on eHealth technology acceptance was non‐significant (path c': *β* = −0.04, SE = 0.03, *p* = 0.18). These results confirm a full mediation effect, indicating that eHealth literacy fully accounted for the relationship between social support and eHealth technology acceptance. This means that social support influences eHealth technology acceptance through its effect on eHealth literacy (Figure 1).

## 4 Discussion

This study provides new evidence from a non‐Western, collectivist cultural context, demonstrating that eHealth literacy fully mediates the relationship between social support and eHealth technology acceptance. Social support alone did not directly influence eHealth adoption unless it contributed to enhancing eHealth literacy, underscoring eHealth literacy as a pivotal mechanism for meaningful engagement with digital health technologies. Additionally, demographic factors such as age, education, occupation, religion and perceived health status were significantly associated with eHealth technology acceptance, consistent with prior research on the role of sociodemographic characteristics in shaping digital health adoption (Huang 2019; Reiners et al. 2019). By examining these relationships in an East Asian collectivist society, this study extends understanding of how social and individual‐level factors interact to influence eHealth engagement, offering insights applicable to digital health strategies in culturally diverse and immigrant populations worldwide.

The results indicate that eHealth technology acceptance among this study's participants is moderate, with notable differences across demographic subgroups. Previous studies have reported that younger and middle‐aged adults are more likely to adopt eHealth technologies due to their familiarity with digital tools (Yang et al. 2020). Our study found that participants aged 41–54 years exhibited the highest eHealth technology acceptance, potentially reflecting a balance between digital proficiency and heightened health management needs (Jeong and Nam 2024). Older adults demonstrated lower initial adoption, often due to limited digital skills or lack of personal devices, but once engaged, they tended to maintain higher adherence than younger users (Reiners et al. 2019). Educational attainment was also a significant predictor, with higher acceptance among those holding college or university degrees, supporting evidence that greater education facilitates both interest in and ability to evaluate and use digital health technologies (Dixit 2018; Paccoud et al. 2021).

Participants with higher social support scores reported greater eHealth literacy, which aligns with studies showing that strong social networks facilitate digital engagement by providing assistance, encouragement and resources (Estrela et al. 2023; Yang et al. 2020). However, in this study, social support alone was insufficient to drive eHealth technology adoption, highlighting the need for integrated strategies combining social support with eHealth literacy training to ensure the effective use of digital health technologies. Among the key predictors examined, eHealth literacy emerged as the strongest predictor, aligning with research indicating that higher eHealth literacy enhances confidence, perceived ease of use and health‐related knowledge (Busse et al. 2022; Kaklamanou et al. 2024). Moreover, social support was insufficient to directly increase eHealth technology acceptance. Social support influenced eHealth acceptance only indirectly by fostering eHealth literacy.

The cultural context offers an important explanation for this mediation effect. In Taiwan and other collectivist East Asian societies, health‐related decisions are often shaped by guidance from family members or trusted healthcare professionals, rather than autonomous personal choice (Chen et al. 2018; Cheng et al. 2024; Li et al. 2021). Trust in healthcare providers and family recommendations plays a crucial role in shaping attitudes toward new interventions (Chu et al. 2024), and patients commonly rely on these significant others to evaluate and explain digital health technologies, rather than independently seeking digital information (Cheng et al. 2024; Kim et al. 2023; Lee and Tak 2022). In collectivist cultures, maintaining harmony and close social ties is highly valued (Felstad 2020), and positive endorsements from trusted figures—such as emphasising ease of use or demonstrating practical benefits—can substantially increase acceptance (Yang Meier et al. 2020). However, without sufficient eHealth literacy, patients may still face challenges in navigating, interpreting and applying digital health information, limiting sustained engagement. This contrasts with Western societies, where adoption is more often driven by perceived personal benefits and autonomous exploration (An et al. 2021). These findings suggest that in collectivist populations, interventions should integrate eHealth literacy skill‐building with the involvement of trusted social structures—such as family networks, community organisations and healthcare providers—to support both uptake and long‐term use.

This study found that eHealth literacy fully mediates the relationship between social support and eHealth technology acceptance, meaning social support influences eHealth adoption only by enhancing eHealth literacy. While social support provides encouragement and exposure to digital health tools, eHealth literacy ultimately facilitates successful adoption. These results highlight the importance of eHealth literacy as a crucial mechanism linking social support to meaningful engagement with eHealth technologies. An explanation for this mediation effect is in the nature of social support and its role in digital health adoption. Although family members, friends and healthcare providers may introduce and encourage eHealth use, patients with low eHealth literacy may still struggle to navigate, interpret and effectively use these technologies (Arcury et al. 2020; De Main et al. 2022). Prior research suggests that social support is an external motivator. However, actual technology engagement requires self‐efficacy and confidence in using digital tools, which are developed through eHealth literacy training and experience (Shi et al. 2024). Without sufficient eHealth literacy, individuals may perceive eHealth platforms as complex, unreliable, or difficult to integrate into their self‐care routines, reducing their likelihood of adoption.

These findings align with the Technology Acceptance Model (TAM), which identifies perceived usefulness and ease of use as primary drivers of technology adoption (Davis et al. 1989). eHealth literacy strengthens these perceptions by equipping individuals with the skills and confidence to evaluate, interpret and integrate digital health tools into their daily routines (Melhem et al. 2023). Without adequate eHealth literacy, even strong social support may not translate into adoption, as individuals may perceive digital platforms as complex, unreliable, or difficult to integrate into self‐care routines. This underscores that social encouragement, while valuable, must be complemented by strategies explicitly targeting eHealth literacy enhancement.

The mediating effect of eHealth literacy may be particularly salient in collectivist cultural contexts, such as Taiwan or other East Asian societies, where health decisions are often influenced by family members or healthcare professionals rather than by autonomous individual choice (Cheng et al. 2024; De Main et al. 2022). In such contexts, patients commonly seek approval or guidance from trusted social figures before engaging with unfamiliar technologies. However, this reliance on social support may not be sufficient if patients lack the foundational literacy needed to independently navigate digital health environments. Therefore, even in the presence of strong social encouragement, insufficient eHealth literacy may limit meaningful engagement. Unlike in Western societies, where individuals may independently explore and adopt new digital health solutions, individuals in Chinese cultural settings tend to seek reassurance and approval from their social network before engaging with unfamiliar eHealth tools (Yang Meier et al. 2020). This underscores the need for culturally tailored eHealth interventions that go beyond merely providing access or offering social encouragement. Instead, interventions must simultaneously address skill‐building and confidence development, leveraging trusted social networks not only for motivation, but also for guided learning. Embedding digital literacy training within existing social structures–such as family support groups, community centres or outpatient clinics–may enhance the sustainability of eHealth adoption in populations with strong collectivist values.

Overall, this study extends existing literature by demonstrating, in a collectivist cultural context, that eHealth literacy is a pivotal mechanism linking social support to eHealth technology acceptance. The findings highlight the need for culturally tailored interventions that combine eHealth literacy training with social support strategies to improve digital health adoption. Such approaches have potential applicability beyond Taiwan, offering guidance for developing inclusive digital health programmes in culturally diverse and immigrant communities worldwide.

### 4.1 Limitations of the Study

This study has several limitations that must be acknowledged. First, the use of a cross‐sectional design limits the ability to establish causal relationships (Hess 2023) among social support, eHealth literacy and eHealth technology acceptance. Second, purposive sampling was employed to recruit individuals with relevant chronic conditions and potential exposure to digital health tools. While this approach enhanced the feasibility and relevance of data collection, it may have introduced selection bias (Tajik et al. 2024), as participants who were more digitally engaged or motivated might have been more inclined to participate. This could limit the generalisability of the findings to broader chronic illness populations. Third, the reliance on self‐reported data introduces potential biases (Bauhoff 2024). Participants might have overestimated or underestimated their own eHealth literacy or technology acceptance due to social desirability, recall inaccuracies, or subjective interpretations of survey items. Additionally, the generalisability of the mediation effect identified in this study may be limited by the specific cultural context. This research was conducted within a predominantly Chinese collectivist culture, where social support from family and healthcare providers significantly shapes health‐related behaviours and technology adoption decisions. Consequently, the observed relationships might differ in more individualistic or culturally diverse populations. Lastly, this study did not account for individual differences in prior experience or familiarity with technology, factors that could significantly influence eHealth acceptance.

### 4.2 Recommendation for Further Research

Although mediation analyses provide valuable insights into associations between variables, future longitudinal studies are needed to elucidate how these relationships evolve and confirm causal directions. Moreover, future research could include objective assessments, such as real‐world usability testing or digital skills assessments, to complement self‐report measures and improve the accuracy of the findings. Additionally, replicating in different cultural contexts would test the stability and applicability of these findings internationally. In the meantime, participants' prior exposure to technology, personal preferences and comfort with digital solutions were systematically considered, as these factors may moderate the relationship between social support, eHealth literacy and technology acceptance.

### 4.3 Implications for Practice

The findings of this study highlight eHealth literacy as a critical mechanism linking social support to digital technology acceptance among patients with chronic illnesses. For healthcare professionals, this suggests that digital interventions should not only consider patients' access to supportive relationships but also assess and enhance their readiness to engage with eHealth technologies.

Routine clinical care should incorporate eHealth literacy screening as a standard practice, particularly when introducing new digital tools for chronic disease management. Tailored interventions—such as peer‐led workshops, digital navigation coaching, or interactive training modules—can foster essential competencies while promoting patient confidence. In parallel, engaging patients' social networks as co‐participants in eHealth learning processes may provide additional reinforcement and reduce anxiety around technology use.

Designers and implementers of eHealth systems must also consider usability, accessibility and cultural relevance when developing platforms for chronic care. Simplified user interfaces, language localisation and responsive design features can increase inclusiveness. Ultimately, a dual‐focus approach that simultaneously strengthens individual capability and mobilises social support holds promise for achieving broader, more equitable uptake of digital health innovations in chronic disease care.

## 5 Conclusions

This study underscores the pivotal role of eHealth literacy in enabling digital health adoption among patients with chronic diseases. In Chinese society, where relationship‐based connections are deeply valued, enhancing patients' eHealth literacy through social support from significant others—such as family members, peers and trusted healthcare professionals—can foster sustained engagement with digital health technologies. Clinically, these findings highlight the need for integrating structured eHealth literacy training with culturally sensitive social support strategies to maximise technology uptake and continued use. From a policy perspective, embedding eHealth literacy development into community health programmes and chronic disease management frameworks may help reduce disparities in digital health participation and improve long‐term health outcomes. Future research should focus on evaluating the sustained effectiveness of culturally tailored, integrated interventions and exploring their transferability and impact across different cultural contexts.

## Author Contributions

Ms. Lian‐Shin Shiu worked on the development of the research framework, methodology, formal analysis and writing the original draft preparation. Miss Yu‐Shan Huang and Miss Yu‐Shan Cheng worked on data collection and implementation of the study. Dr. Chieh Yu Liu carried out the analyses, and wrote and revised the manuscript. Dr. Yu‐Chi Chen worked on the development of the research framework, formal analysis and investigation, writing the original draft preparation, review and editing. All authors reviewed and approved the manuscript prior to submission.

## Conflicts of Interest

The authors declare no conflicts of interest.

## Supporting information


**Data S1:** jan70207‐sup‐0001‐DataS1.pdf.

**Data S2:** jan70207‐sup‐0002‐DataS2.pdf.

## Data Availability

The data that support the findings of this study are available from the corresponding author upon reasonable request.
