# Supplementary material for: eHealth Literacy Mediating Social Support and Technology Acceptance Among Patients With Chronic Illnesses: A Cross‐Sectional Study
Source: J Adv Nurs. 2025 Sep 8;82(5):5049–59. doi: 10.1111/jan.70207 (PMC13069186; doi:10.1111/jan.70207)
Supplement: Supplementary file 1 — Data S1: jan70207‐sup‐0001‐DataS1.pdf. [file JAN-82-5049-s002.pdf]

Supplementary 1.　The eHealth Literacy and eHealth Acceptance scales

1. Chinese version of eHealth Literacy Questionnaire scale names and items[a]

1.運用科技來處理健康資訊	
	我使用科技找……
	我經常使用科技……
	科技幫助我決定怎樣……
	我使用科技分享…….
	我使用科技來……
2.了解健康概念和語言	
	我具備的知識有助於……
	關於我的健康我有足夠…..
	我了解關於我自己…….
	整體而言，我了解我的……
	我使用身體的測量…..
3.主動參與數位服務的能力	
	我知道如何使用科技取得……..
	我知道如何受惠於科技……
	我能夠將資料輸入…..
	我很快地學會如何…..
	我很容易學會…..
4.感到安全和控制	
	我確定只有應該使用的人…….
	我的電子健康照護……
	我清楚地了解健康照護人員……
	我確定只有被授權的人……

	我有信心健康照護人員…..
5.被激發參與數位服務	
	科技讓我覺得能主動…….
	我發覺科技可幫助….
	當我使用科技時…..
	科技促進我…..
	我發覺科技有助……
6.取得的數位服務是可用的	
	需要關於我健康資訊的人……
	我的健康照護人員提供的服務……..
	不論我在任何地方……
	所有我使用的健康科技……
	我的大部分醫療照護人員……
	我接觸過的健康科技……
7.數位服務符合個人需求	
	我發覺健康科技服務…..
	我發覺健康科技…..
	我發覺健康科技服務……
	健康科技服務提供……

2. The Chinese version of eHealth Acceptance scale names and items[a]

題號	題目	非常不同意	不同意	同意	非常同意
1	使用健康資訊科技可以增加…………	☐	☐	☐	☐
2	使用健康資訊科技能協助…………	☐	☐	☐	☐
3	健康資訊科技所提供的功能…………	☐	☐	☐	☐
4	使用健康資訊科技可讓我…………	☐	☐	☐	☐
5	使用健康資訊科技能讓我…………	☐	☐	☐	☐
6	使用健康資訊科技使我…………	☐	☐	☐	☐
7	整體而言，使用健康資訊科技對我…………	☐	☐	☐	☐
8	使用健康資訊科技查詢我…………	☐	☐	☐	☐
9	對我來說學會操作健康資訊科技設備…………	☐	☐	☐	☐
10	對我來說學會操作健康資訊科技介面…………	☐	☐	☐	☐
11	我很容易就記住健康資訊科技…………	☐	☐	☐	☐
12	我很容易成為熟練操作健康資訊科技…………	☐	☐	☐	☐
13	我所接觸過的健康資訊科技介面…………	☐	☐	☐	☐
14	我會主動徵詢健康資訊科技…………	☐	☐	☐	☐
15	我會願意嘗試別人建議…………	☐	☐	☐	☐
16	我認為使用健康資訊科技來替代傳統健康管理…………	☐	☐	☐	☐
17	我認為使用健康資訊科技來進行健康管理…………	☐	☐	☐	☐
18	我會特別關注健康資訊科技…………	☐	☐	☐	☐
19	未來的生活中，我會嘗試使用各種不同的健康資訊科技…………	☐	☐	☐	☐
20	我將會持續使用健康資訊科技系統或產品…………	☐	☐	☐	☐
21	我會想使用健康資訊科技…………	☐	☐	☐	☐

[a]Items are truncated. The full list of items is available with the authors.
