# Supplementary material for: eHealth Literacy Mediating Social Support and Technology Acceptance Among Patients With Chronic Illnesses: A Cross‐Sectional Study
Source: J Adv Nurs. 2025 Sep 8;82(5):5049–59. doi: 10.1111/jan.70207 (PMC13069186; doi:10.1111/jan.70207)
Supplement: Supplementary file 2 — Data S2: jan70207‐sup‐0002‐DataS2.pdf. [file JAN-82-5049-s001.pdf]

Run MATRIX procedure:

**************** PROCESS Procedure for SPSS Version 4.2 ****************

Written by Andrew F. Hayes, Ph.D. www.afhayes.com

Documentation available in Hayes (2022). www.guilford.com/p/hayes3

****

Model : 4

Y : eHealth technology acceptance

X : social support

M : eHealth literacy

Covariates : Age, education, occupation, religion, perceived health status

Sample Size: 161

****

OUTCOME VARIABLE:

eHealth literacy

Model Summary

R	R-sq	MSE	F	df1	df2	p
529	.3057	140.6860	11.3018	6.0000	154.0000	.0000

Model

	coeff	se	t	p	LLCI	ULCI
constant	78.4814	7.1345	11.0002	.0000	64.3872	92.5756
social support	.2922	.0606	4.8189	.0000	.1724	.4120
Age	-2.3982	.8978	-2.6711	.0084	-4.1718	-.6245
education	2.4546	1.0089	2.4330	.0161	.4616	4.4477
occupation	4.9222	2.1071	2.3360	.0208	.7597	9.0847
religion	-.9383	1.0008	-.9375	.3499	-2.9153	1.0388
perceived health status	-1.1207	1.5929	-.7035	.4828	-4.2674	2.0261

****

OUTCOME VARIABLE:

eHealth technology acceptance

Model Summary

R	R-sq	MSE	F	df1	df2	p
.7923	.6278	30.2843	36.8642	7.0000	153.0000	.0000

Model

	coeff	se	t	p	LLCI	ULCI
constant	9.0533	4.4234	2.0467	.0424	.3144	17.7922
social support	-.0409	.0302	-1.3565	.1769	-.1006	.0187
eHealth literacy	.4940	.0374	13.2129	.0000	.4201	.5679
Age	.0213	.4261	.0500	.9602	-.8205	.8631
education	.1044	.4770	.2188	.8271	-.8380	1.0467
occupation	.6597	.9948	.6632	.5082	-1.3055	2.6250
religion	.6712	.4657	1.4414	.1515	-.2488	1.5911
perceived health status	1.2187	.7402	1.6463	.1017	-.2437	2.6810

************************** TOTAL EFFECT MODEL ********************

OUTCOME VARIABLE:

eHealth technology acceptance

Model Summary

R	R-q	MSE	F	df1	df2	p
.4506	.2031	64.4191	6.5400	6.0000	154.0000	.0000

Model

	coeff	se	t	p	LLCI	ULCI
constant	47.8226	4.8278	9.9057	.0000	38.2853	57.3598
social support	.1034	.0410	2.5202	.0127	.0224	.1845
Age	-1.1634	.6075	-1.9149	.0574	-2.3636	.0368
education	1.3170	.6827	1.9291	.0556	-.0317	2.6656
occupation	3.0913	1.4258	2.1681	.0317	.2746	5.9079
religion	.2077	.6772	.3067	.7595	-1.1302	1.5455
perceived health status	.6651	1.0779	.6170	.5381	-1.4642	2.7944

************** TOTAL, DIRECT, AND INDIRECT EFFECTS OF X ON Y *****

Total effect of X on Y

Effect	se	t	p	LLCI	ULCI
.1034	.0410	2.5202	.0127	.0224	.1845

Direct effect of X on Y

Effect	se	t	p	LLCI	ULCI
-.0409	.0302	-1.3565	.1769	-.1006	.0187

Indirect effect(s) of X on Y:

	Effect	BootSE	BootLLCI	BootULCI
eHealth literacy	.1444	.0305	.0874	.2068

********************** ANALYSIS NOTES AND ERRORS ***************

Level of confidence for all confidence intervals in output:

95.0000

Number of bootstrap samples for percentile bootstrap confidence intervals:

1000

------ END MATRIX -----
